# Efficacy of probiotic supplementation for body weight management in overweight and obese adults: a meta-analysis of randomized controlled trials predominantly from East Asia

**DOI:** 10.3389/fpubh.2026.1767108

**Published:** 2026-03-20

**Authors:** Shuying Liu, Kani Ouyang, Xuelian Fang, Huijuan He, Mengying Li

**Affiliations:** 1 School of Nursing, Hubei University of Chinese Medicine, Wuhan, Hubei, China; 2 Affiliated Hospital of Hubei University of Chinese Medicine, Wuhan, Hubei, China

**Keywords:** meta-analysis, obesity, probiotics, public health, weight management

## Abstract

**Background:**

Obesity is a major risk factor for multiple chronic diseases. Probiotics, as a novel intervention strategy for obesity, exhibit varying efficacy depending on factors such as strain, dosage, and duration of administration. This variability leads to inconsistent existing conclusions, hindering their standardized application.

**Methods:**

Seven electronic databases were searched from their inception to August 2025. The Cochrane RoB 1.0 tool was used to assess the quality of included randomized controlled trials. Statistical analysis was performed using Review Manager 5.4, and sources of heterogeneity were explored through subgroup analysis.

**Results:**

A meta-analysis of 12 randomized controlled trials demonstrated that probiotics effectively improve body weight (MD = −0.52, 95% CI − 0.90 to −0.13, *p* < 0.01), BMI (MD = −0.22, 95% CI − 0.34 to −0.11, *p* < 0.01), waist circumference (MD = −0.29, 95% CI − 0.56 to −0.02, *p* = 0.03), and body fat percentage (MD = −0.59, 95% CI − 0.96 to −0.21, *p* < 0.01) in obese patients, but it has no significant effect on total cholesterol (MD = −2.94, 95% CI − 6.05 to 0.17, *p* = 0.06) and triglycerides (MD = −6.77, 95% CI (−14.58 to 1.05, *p* = 0.09). Among these, regimens using single strains at high doses (≥1 × 10^10^ CFU/day) or combined with health guidance yielded more pronounced effects in individuals with simple obesity.

**Conclusion:**

Probiotic supplementation has a positive effect on reducing body weight, BMI, waist circumference, and body fat percentage in overweight and obese adults; however, it does not show a significant improvement in blood lipid indicators. Subgroup analysis indicates that single-strain, high-dose, and probiotic intervention plus health guidance are more effective, suggesting that probiotic interventions are both strain-specific and outcome-specific. Future large-scale randomized controlled trials should focus on strain specificity, long-term intervention effects, and cost-effectiveness.

**Systematic review registration:**

Publicly accessible website: https://www.crd.york.ac.uk/PROSPERO/view/CRD420251123057, Registration number: CRD420251123057.

## 1 Introduction

The global rise in overweight and obesity is closely associated with shifts in lifestyle and dietary patterns (1, 2). It is projected that by 2030, the prevalence of overweight and obesity among Chinese adults will reach 61% (3). Obesity not only modifies body morphology but also markedly elevates the risk of type 2 diabetes mellitus (T2DM), cardiovascular disease, metabolic dysfunction-associated steatotic liver disease (MASLD) (4–6), and specific malignancies by instigating pathological mechanisms such as chronic low-grade inflammation and insulin resistance. It concurrently exerts significant adverse effects on patients’ mental health and quality of life, imposing considerable pressure on healthcare systems (7). In light of this, identifying safe, effective, and sustainable strategies for long-term weight management has become a priority in both public health initiatives and clinical practice.

Recent research on gut microbiota has yielded novel insights into the prevention and treatment of obesity. Evidence indicates that gut microbiota and their metabolites exert significant anti-obesity effects by modulating intestinal immune responses and maintaining homeostasis (8, 9). The WHO definition of a probiotic–“live microorganisms which when administered in adequate amounts confer a health benefit on the host”. Common strains in weight management mainly include Lactobacillus and Bifidobacterium. The weight loss and lipid-lowering effects of probiotics may mainly be achieved through the following two mechanisms: first, lactobacilli can regulate the structure and diversity of the gut microbiota; second, various metabolites of lactobacilli can participate in the host’s metabolic regulation and produce positive effects (10, 11). Currently, several clinical studies support the positive role of specific probiotic strains in weight management. For example, a randomized controlled trial in obese individuals found that intervention with *Lactobacillus plantarum* significantly reduced participants’ body fat (12). Wang’s (13) interventional study found that probiotics did not show a clear weight-loss aiding effect in post-bariatric surgery populations, which may be related to strain specificity, intervention duration, and differences in participants’ metabolic status; in addition, some studies suggest that combining probiotics with exercise interventions can produce a synergistic effect, further improving metabolic indicators (14). These inconsistent results suggest that the weight-loss effects of probiotics are influenced by multiple factors, including the type of strain, dosage, duration of intervention, and whether combined with lifestyle interventions, which limits their standardized application in clinical and public health practice. Therefore, clarifying the independent and synergistic roles of probiotics—especially when integrated with exercise guidance—is crucial for advancing comprehensive obesity prevention strategies that holistically combine nutrition, physical activity, and behavioral interventions in public health practice.

To resolve the controversies above, this study aims to employ a meta-analysis methodology to systematically identify and synthesize published randomized controlled trials worldwide that investigate probiotic interventions for weight management in obese patients. Following a rigorous quality assessment of the included studies, a pooled effect size analysis will be performed to evaluate the overall efficacy of probiotics in weight control for obese individuals. Further, this study will thoroughly examine potential effect variations related to different probiotic strains, dosages, treatment durations, and adjunctive interventions. This work intends to generate high-quality evidence to inform the future clinical application of probiotics in obesity management and to provide a foundation for guiding subsequent research priorities.

## 2 Methods

This study’s design and conduct precisely followed the methodological framework for systematic reviews and meta-analyses set out by the Joanna Briggs Institute (JBI). Reporting follows the Preferred Reporting Items for Systematic Review and Meta-analyses (PRISMA) guidelines. The research protocol was registered in the PROSPERO platform under registration number CRD420251123057.

### 2.1 Search strategy

A systematic literature search was conducted across seven electronic databases, including China National Knowledge Infrastructure (CNKI), Wanfang Data, VIP, PubMed, Embase, the Cochrane Library, and Web of Science. The search encompassed all records from the inception of each database up to August 2025. The search strategy was developed using the PICO (Population, Intervention, Comparison, Outcome) framework and combined Medical Subject Headings (MeSH) with free-text terms, linked by Boolean operators (AND, OR). The aim was to identify studies investigating the effects of probiotic interventions on weight management in individuals with obesity. Key search terms included ‘probiotics,’ ‘obesity,’ and ‘body weight’. The detailed search strategies are provided in Supplementary Table S1.

### 2.2 Inclusion criteria

The inclusion criteria were established based on the PICOS framework. Studies fulfilling all the following criteria were eligible for inclusion in this review:

1. Study Design: Randomized controlled trials (RCTs).
2. Study Population: Adult participants (aged ≥18 years) with overweight or obesity, defined according to the World Health Organization’s criteria for Asian populations as a body mass index (BMI) of ≥23 kg/m^2^ (overweight) or ≥27.5 kg/m^2^ (obese) (15); Individuals using weight-loss, lipid-lowering, or glucose-lowering medications were excluded. No gender restrictions were applied.
3. Intervention: The experimental group received a defined probiotic intervention (single or multi-strain), with specification of strain(s), dosage, and route of administration (e.g., capsules, powder, or fermented dairy products). The intervention duration was required to be ≥4 weeks. The control group received a placebo or no active intervention.
4. Outcome Measures: The primary outcomes were changes in body weight (kg), BMI (kg/m^2^), and body fat percentage (%). Secondary outcomes included waist circumference, serum triglyceride levels, and total cholesterol levels.

### 2.3 Exclusion criteria

Studies were excluded from this review if they met any of the following criteria:

1. Duplicate publications.
2. Non-randomized controlled studies.
3. Studies with inadequate outcome data or data that could not be transformed or extracted for meta-analysis.
4. Studies for which the full-text article was unavailable.
5. Studies were assessed as having an overall high risk of bias, specifically those with two or more domains rated as ‘high risk’ according to the Cochrane Risk of Bias tool.

### 2.4 Literature screening and data extraction

Two researchers independently conducted literature screening based on the predetermined inclusion and exclusion criteria. Using the reference management software EndNote 21, they initially reviewed titles and abstracts to exclude papers that evidently did not satisfy the eligibility criteria. Following this preliminary screening, full-text papers were assessed to exclude non-compliant studies. Consistency checks were performed throughout the process, and any discrepancies between reviewers were resolved through discussion or, when necessary, consultation with a third researcher to reach a final decision on inclusion. The extracted data pieces encompassed fundamental study information, participant demographics, intervention strategies, and outcome measures.

### 2.5 Quality evaluation

The methodological quality of the included studies was assessed using the Cochrane Risk of Bias Tool (RoB 1.0) (16) and Review Manager 5.4 software. Prior to formal assessment, two reviewers collaboratively established a preset table of risk of bias judgment criteria (Supplementary Table S2) in accordance with the guiding principles of the Cochrane Handbook (RoB version 1.0), which served as a consistent foundation throughout the evaluation process. The assessment covered the following domains: ① Selection bias; ② Performance bias; ③ Detection bias; ④ Attrition bias; ⑤ Reporting bias; ⑥ Other bias. Each domain was independently rated by two reviewers as “low risk,” “high risk,” or “unclear risk.” Any disagreements were resolved through discussion.

### 2.6 Statistical methods

A meta-analysis was conducted using Review Manager 5.4 software. A fixed-effect model was applied when statistical homogeneity was present across studies (*p* ≥ 0.1, *I*^2^ ≤ 50%); otherwise, a random-effects model was used. Pre-specified subgroup analyses were conducted to explore potential sources of heterogeneity and determine their causes. Studies were stratified into the following four subgroups according to the inclusion criteria: (1) Study Population: simple obesity and overweight/obese; (2) Intervention Dose: high-dose group (≥1 × 1,010 CFU/day) and low-dose group (<1 × 1,010 CFU/day), this dose threshold is established based on the biological effects that the commonly used doses and bacterial counts in previous studies may exert on host metabolism (17); (3) Number of strains: single-strain and multi-strain formulations; (4) Intervention approach: probiotic-only intervention group and probiotic intervention plus health guidance group (providing dietary and physical activity education to all participants). It should be noted that subgroup analyses with few studies may lack statistical power and limit the robustness of findings. All outcomes were continuous variables. Because weight, BMI, waist circumference, and related markers shared consistent units across trials, treatment effects were expressed as weighted mean differences (WMD) accompanied by 95% confidence intervals (CI). A *p* value of less than 0.05 was deemed statistically significant. Additionally, a sensitivity analysis was conducted by excluding individual studies to assess the stability of the results. For outcomes with 10 or more included studies, publication bias was assessed using funnel plots and Egger’s test.

## 3 Results

### 3.1 Search results

**Figure 1:**
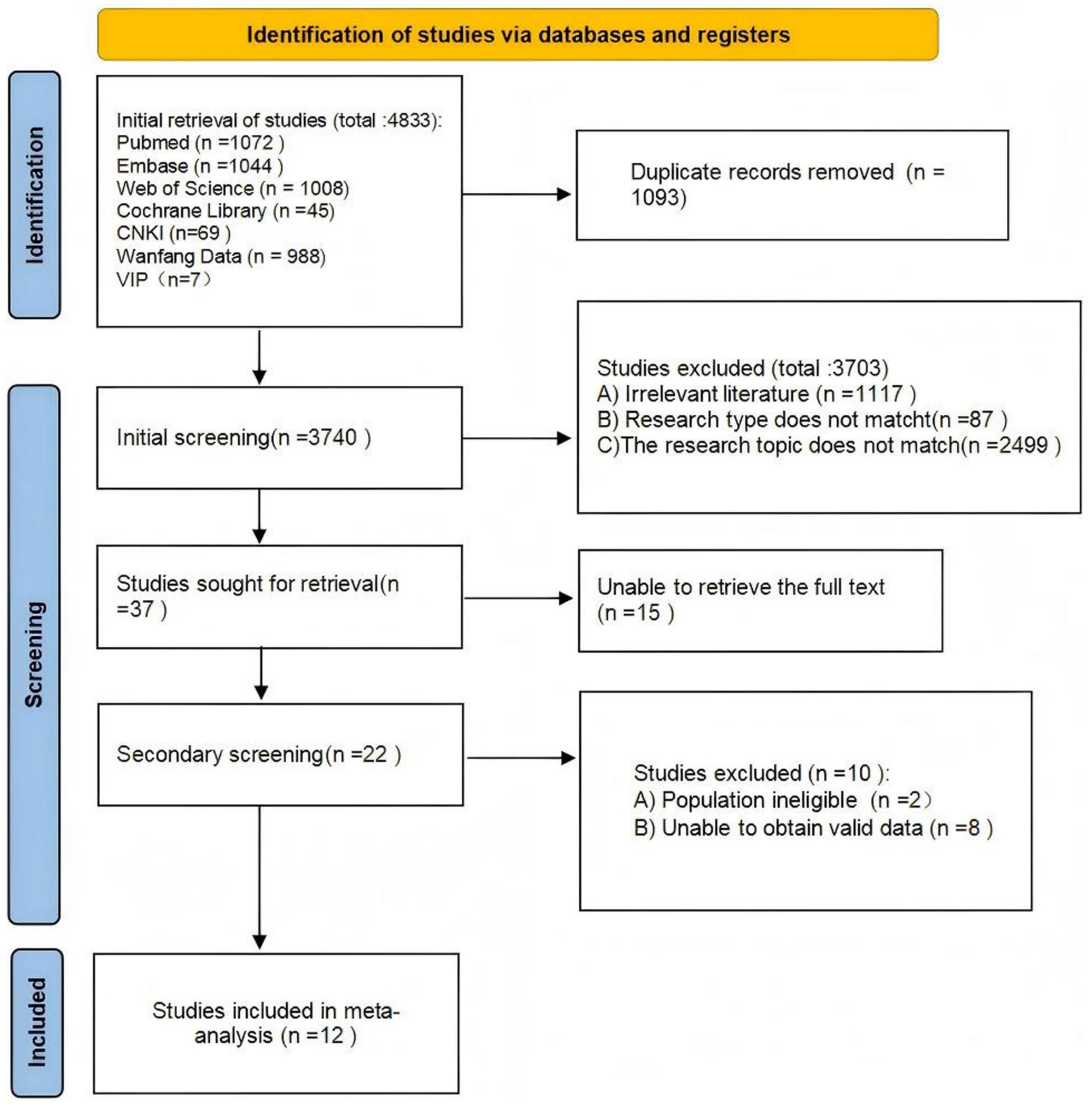
PRISMA flow diagram of study selection process.

The initial systematic database search yielded 4,833 records. After removal of 1,093 duplicates, 3,703 records were excluded based on title and abstract screening against the eligibility criteria. Full-text screening was performed for the remaining 37 articles. Of these, 15 were excluded due to unavailability of full text, and a further 10 were excluded after assessment: 2 due to mismatched research populations and 8 due to insufficient valid data. Ultimately, 12 studies were included in the meta-analysis. The study selection process is summarized in Figure 1 (PRISMA flow diagram).

### 3.2 Basic characteristics included in the study

Twelve randomized controlled trials (RCTs) were included in the meta-analysis (12, 18–28). Geographically, most studies were conducted in Asia: 10 in South Korea, two in China, and one in Saudi Arabia. All trials had an intervention duration of 12 weeks. The probiotics used belonged primarily to the *Lactobacillus genus* (*Lactobacillus plantarum* and *Lactobacillus gasseri*), as well as to the *Bifidobacterium* and *Weissella genera*; some studies employed multi-strain combinations of *Lactobacillus* and *Bifidobacterium*. Daily doses ranged from 1 × 10^8^ CFU to 5 × 10^10^ CFU, with 1 × 10^10^ CFU and 5 × 10^9^ CFU being the most frequently used (four studies each). Administration frequency varied from once to three times daily, with once- and twice-daily regimens being the most common. Detailed study characteristics are presented in Table 1.

**Table 1:** Data extraction sheet.

Study (year)	Country	Participants	Intervention		Intervention duration	Genus	intervention frequency (times/day)	Dosage (times/CFU)	Outcome measure
			T	C					
Sun et al. (28)	South Korea	40/34	Oral *WCFA19* (containing *WIKIM51*) capsules	Placebo capsules	12 weeks	*Weissezia*	1	1 × 1,010	BW, WC, BMI, TC, TG
Sohn et al. (25)	South Korea	50/49	*Lmt1-48* capsule	Placebo capsules	12 weeks	*Lactobacillus*	2	1 × 1,010	BW, BMI, WC, TC, TG
Sohn, Na (26)	South Korea	41/40	Probiotic capsule *CKDB156*	Placebo capsules	12 weeks	*Lactobacillus*	2	2 × 109	BMI, BW, WC, TC, TG
Song et al. (27)	South Korea	25/25	Oral probiotic capsules containing *B. breve CBT BR3* and *L. plantarum CBT LP3*	Placebo capsules	12 weeks	*Lactobacillus* and *Bifidobacterium*	1	3 × 1,010	BW, WC, BMI, TC, TG
AlMalki et al. (18)	Saudi Arabia	44/49	Oral multi strain probiotic powder (containing 3 kinds of *Lactobacillus* and 3 kinds of *Bifidobacterium*)	Placebo capsules	12 weeks	*Lactobacillus* and *Bifidobacterium*	2	3 × 1,010	BW, BMI, TC, TG
Lee et al. (12)	South Korea	53/53	Oral *L. plantarum lmt1-48* capsules	Placebo capsules	12 weeks	*Lactobacillus*	1	5 × 109	BW, CM, BMI, TC, TG
Mo et al. (24)	South Korea	29/30	Oral capsules *L. curvatuscampylosus HY7601* and *L. plantarum KY1032*	Placebo capsules	12 weeks	*Lactobacillus*	1	1 × 1,010	BW, CM, BMI, BFP, TC, TG
Lim et al. (23)	South Korea	57/57	*Lactobacillus sakei cjls03* (kimchi strain)	Placebo capsules	12 weeks	*Lactobacillus*	2	5 × 109	BW, BMI, WC, TC, TG
Jung et al. (21)	China	28/29	Oral *BNR17* capsules	Placebo capsules	12 weeks	*Lactobacillus*	3	1 × 1,010	BW, CM, BMI, BFP, TC, TG
Cho et al. (19)	South Korea	38/37	Capsules containing two strains of probiotics(*L. fermentum MG4231*and *MG4244*)	Placebo capsules	12 weeks	*Lactobacillus*	1	5 × 109	BFP, BW, TC, TG
Kwon et al. (29)	South Korea	67/58	Oral *BN-202 M* powder(*Lacticaseibacillus paracasei BEPC22* and *Lactiplantibacillus plantarum BELP5*)	Placebo capsules	12 weeks	*Lactobacillus*	1	5 × 1,010	BW, CM, BMI, BFP, TC, TG
Chu et al. (20)	China	18/13	*L. bulgaricus* powder	Placebo capsules	12 weeks	*Lactobacillus*	1	1 × 108	BW, CM, BMI, BFP, TC, TG

T, Test Group, C, Control Group, BW, Body Weight, WC, Waist Circumference, BMI, Body Mass Index, BFP:body fat percentage, TC, Total Cholesterol, TG, Triglyceride.

### 3.3 Quality evaluation of included studies

**Figure 2:**
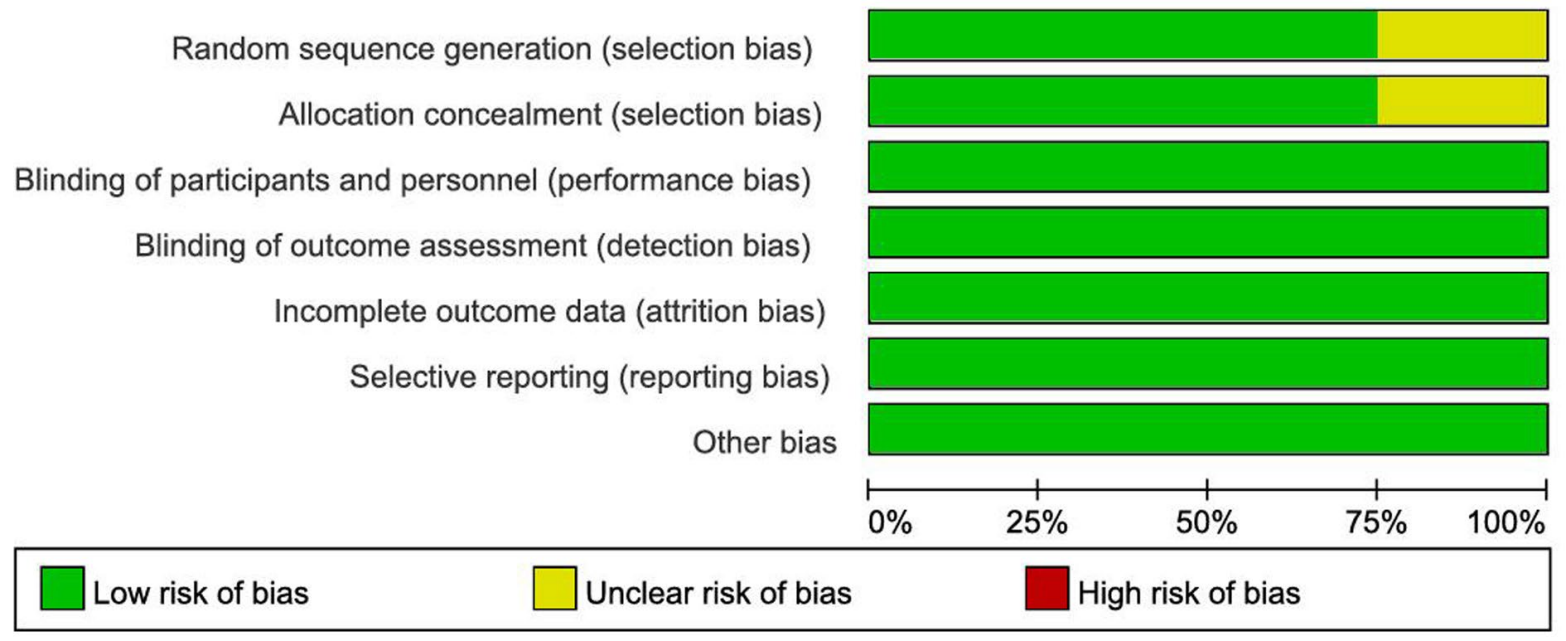
Risk of bias assessment.

**Figure 3:**
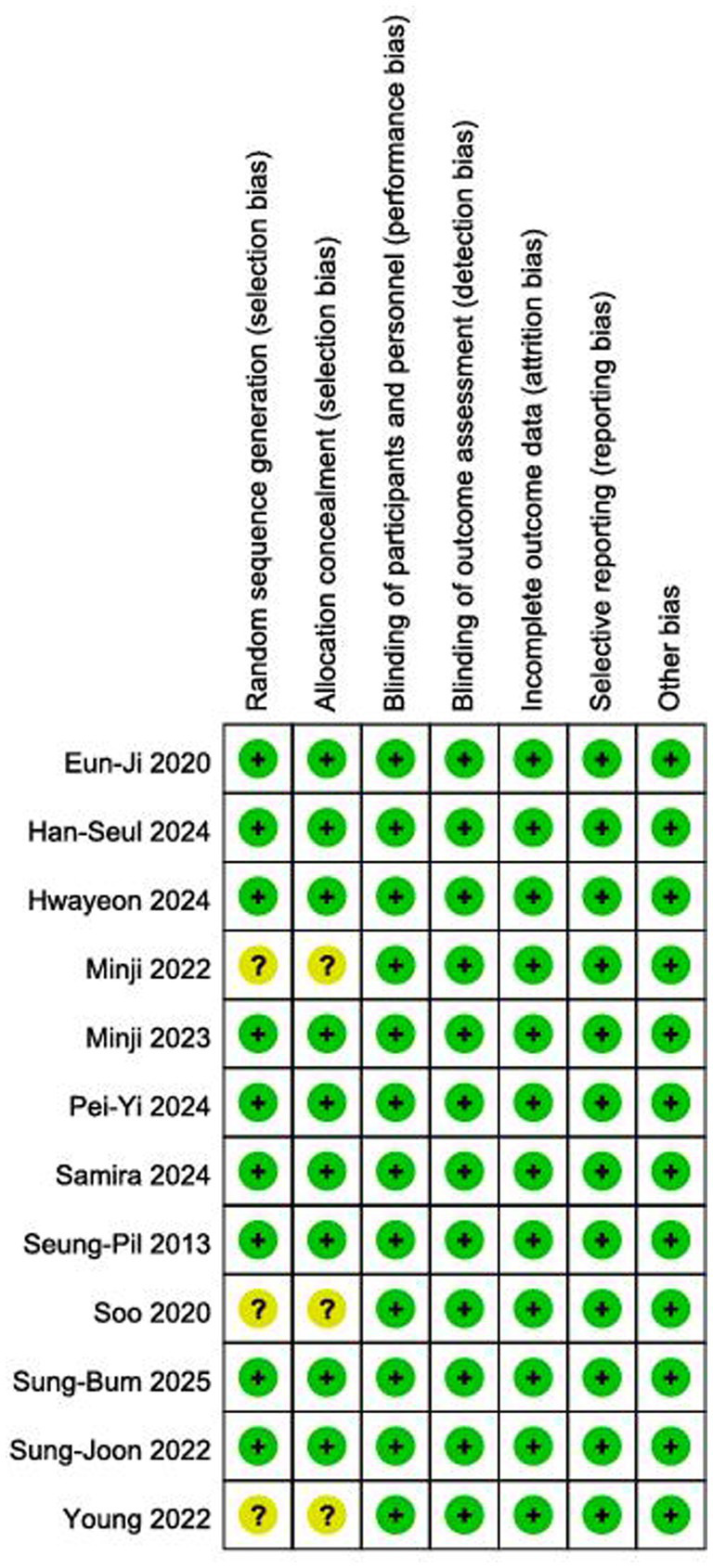
Summary of bias risk assessment.

The methodological quality of the 12 included studies was assessed using the Cochrane Risk of Bias tool (version 1.0). The results are presented in Figures 2, 3. Overall, the included studies were rated as having moderate to high quality. The assessment for each domain is summarized as follows: (1) Random sequence generation and allocation concealment: Nine studies described using random number tables or computer-generated randomization, which were classed as low risk; three studies did not describe randomization methods and were graded as unclear risk. Nine trials reported adequate methods and were considered low risk, while three provided insufficient information and were rated as unclear risk. (2) Blinding implementation: All 12 trials were classified as low-risk. (3) Incomplete outcome data and selective reporting: All 12 trials reported pre-specified outcome measures without selective reporting, indicating low risk. (4) Other biases: No sources of bias influencing study outcomes were found in any of the 12 investigations, indicating a low risk. Although some studies lacked details on randomization and concealment, all trials demonstrated rigorous blinding, complete outcome reporting, and adherence to pre-specified outcomes, which supports the reliability of the findings.

### 3.4 Meta-analysis results

#### 3.4.1 Impact on body weight

**Figure 4:**
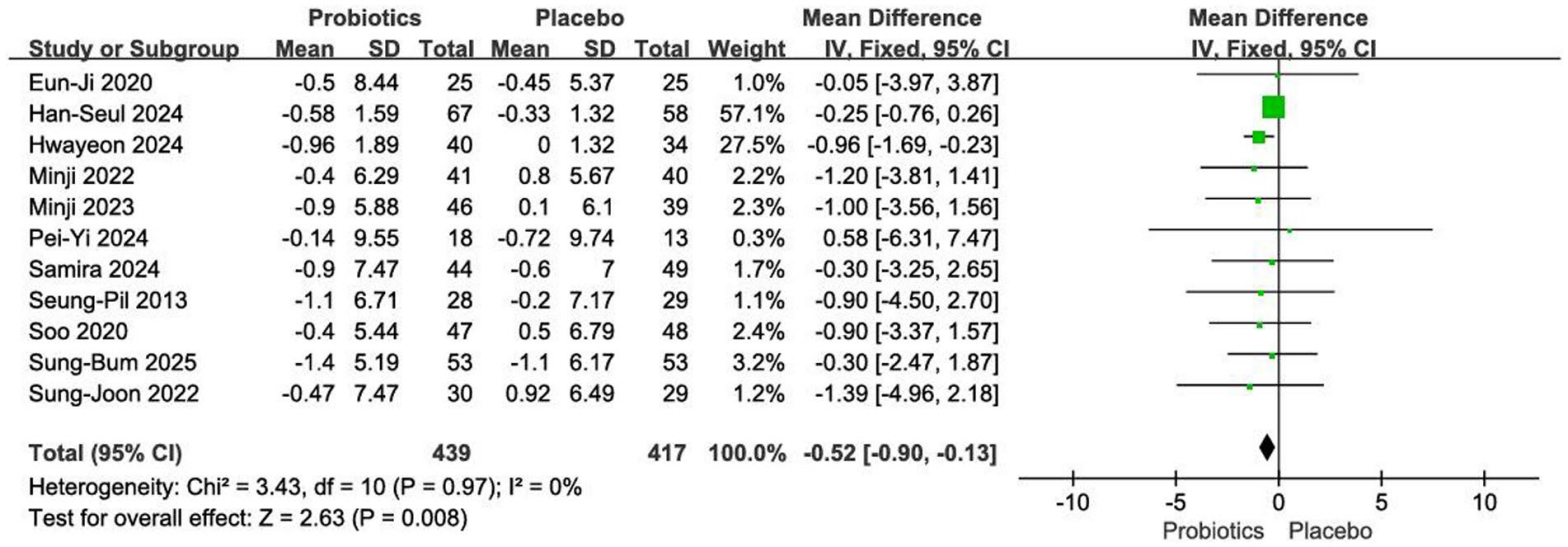
Forest plot of meta-analysis on body weight.

A total of 856 participants were enrolled across the 11 included RCTs (12, 18, 20–28). Given the low heterogeneity among studies (*I*^2^ = 0%, *p =* 0.97), a fixed-effects model was applied. The meta-analysis showed that probiotic intervention significantly reduced body weight compared with placebo [MD = −0.52 kg, 95% CI (−0.90, −0.13), *p* < 0.01] (Figure 4).

#### 3.4.2 Impact on BMI

**Figure 5:**
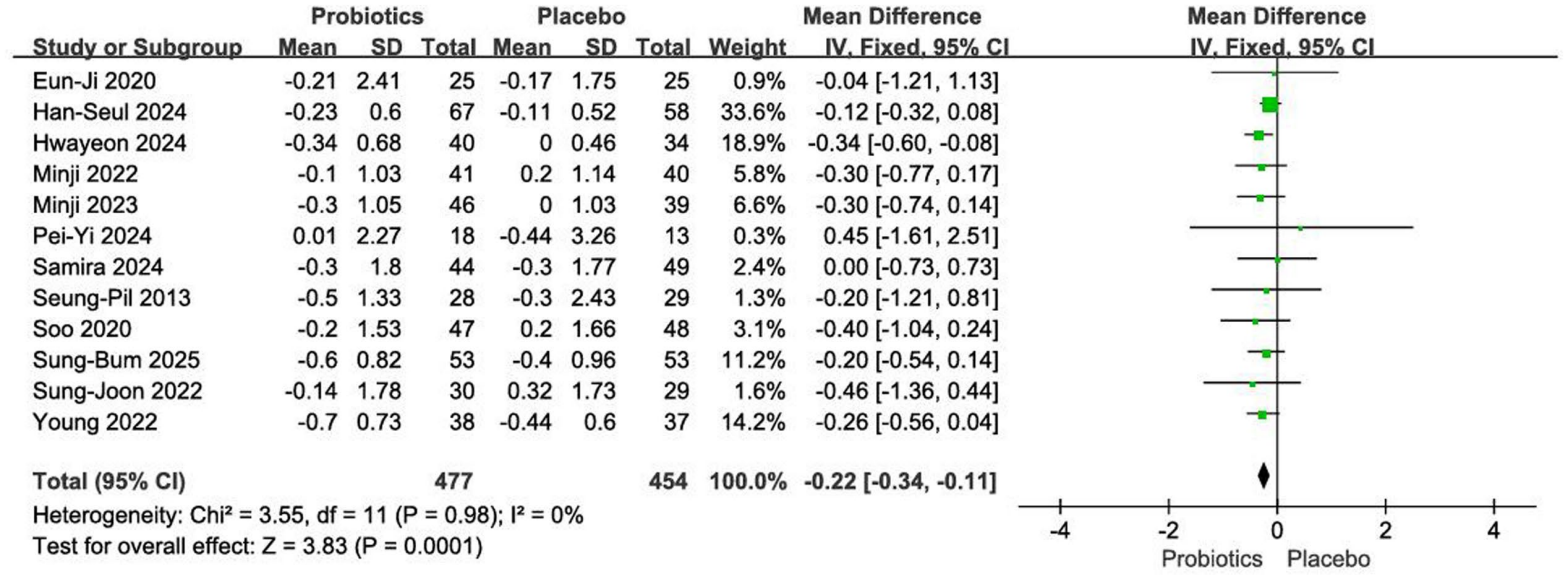
Forest plot of meta-analysis on BMI.

Twelve RCTs involving 931 participants were included in this analysis (12, 18–28). Due to low between-study heterogeneity (*I*^2^ = 0%, *p* = 0.98), a fixed-effects model was used. The pooled results indicated that probiotic intervention led to a significantly greater reduction in BMI compared with placebo [MD = −0.22 kg/m^2^, 95% CI (−0.34, −0.11), *p* < 0.01] (Figure 5).

#### 3.4.3 Impact on waist circumference

**Figure 6:**
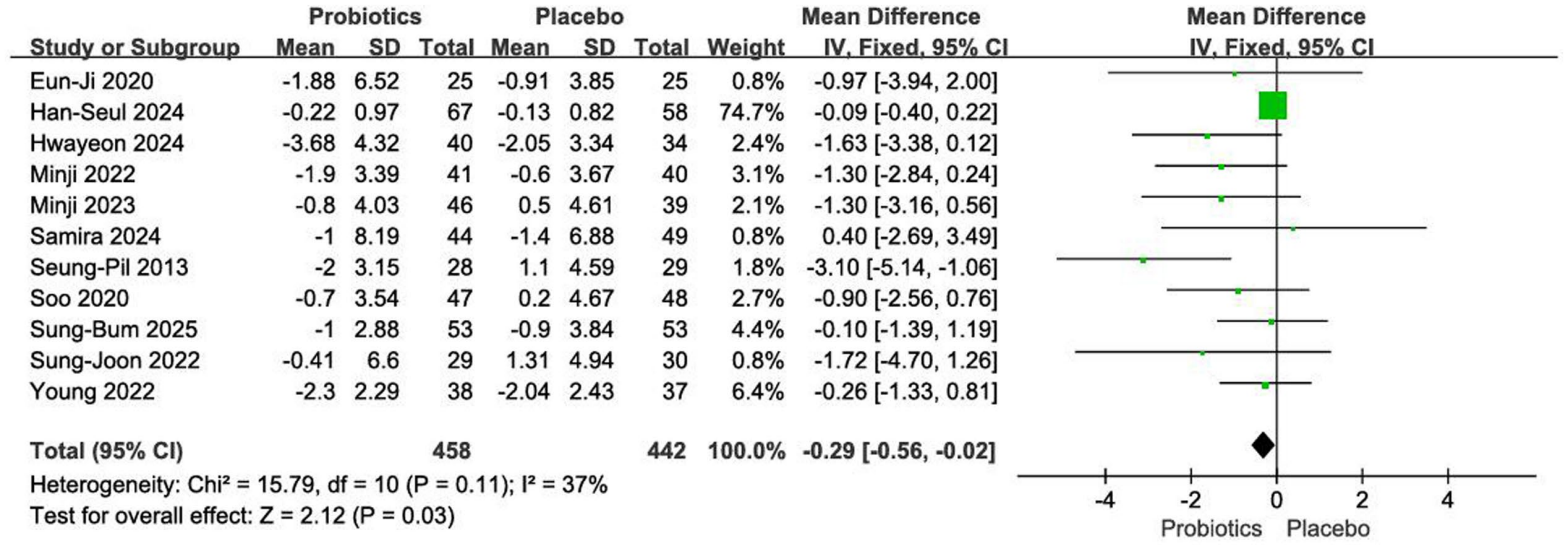
Forest plot of meta-analysis on waist circumference.

Eleven RCTs with a total of 900 participants were analysed (12, 18, 19, 21–28). Given the low between-study heterogeneity (*I*^2^ = 37%, *p* = 0.11), a fixed-effects model was applied. The meta-analysis showed that probiotic intervention significantly reduced waist circumference compared with placebo [MD = −0.29 cm, 95% CI (−0.56, −0.02), *p* = 0.03] (Figure 6).

#### 3.4.4 Impact on body fat percentage

**Figure 7:**
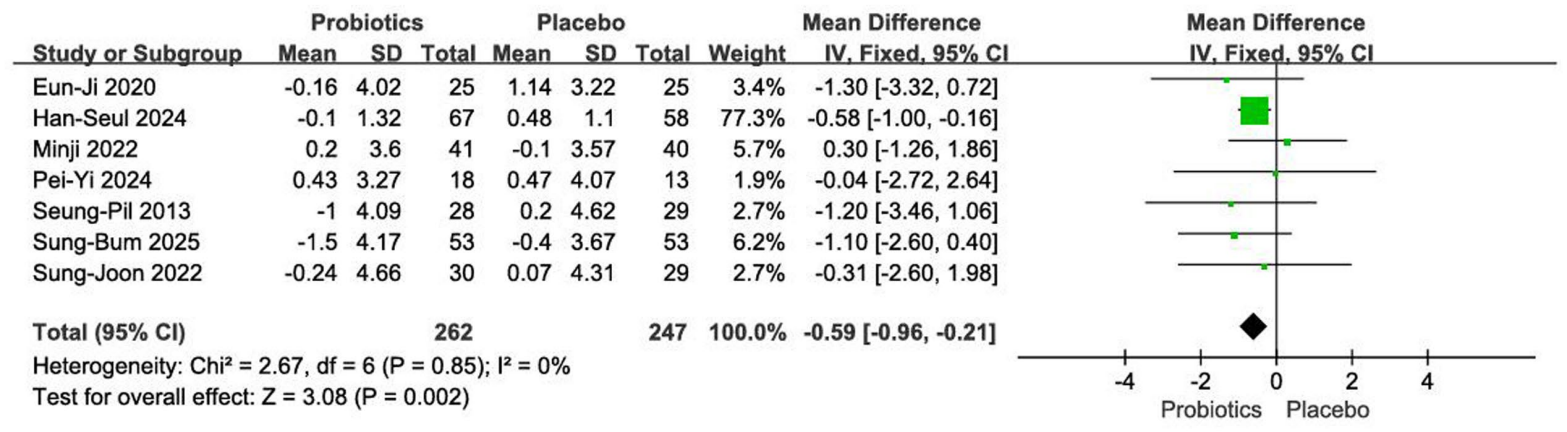
Forest plot of meta-analysis on body fat percentage.

Seven RCTs comprising 509 participants were included in the analysis (12, 20–22, 24, 26, 27). Owing to the low between-study heterogeneity (*I*^2^ = 0%, *p* = 0.85), a fixed-effects model was employed. The pooled results demonstrated a significantly greater reduction in body fat percentage with probiotic intervention compared to placebo [MD = −0.59, 95% CI (−0.96, −0.21), *p* < 0.01] (Figure 7).

#### 3.4.5 Impact on total cholesterol

**Figure 8:**
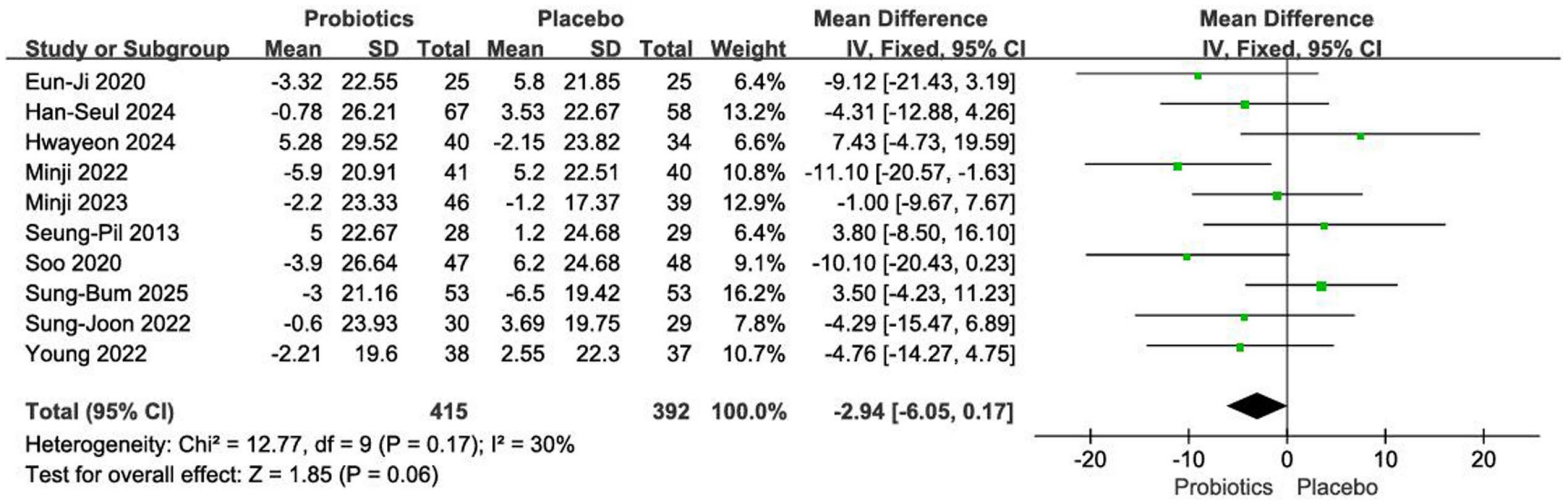
Forest plot of meta-analysis on total cholesterol.

Ten RCTs with a total of 807 participants were analysed (12, 18, 19, 21–28). Given the low between-study heterogeneity (*I*^2^ = 30%, *p* = 0.17), a fixed-effects model was used. The meta-analysis found no statistically significant difference in total cholesterol reduction between the probiotic and placebo groups [MD = −2.94 mg/dL, 95% CI (−6.05, 0.17), *p* = 0.06] (Figure 8).

#### 3.4.6 Impact on triglyceride

**Figure 9:**
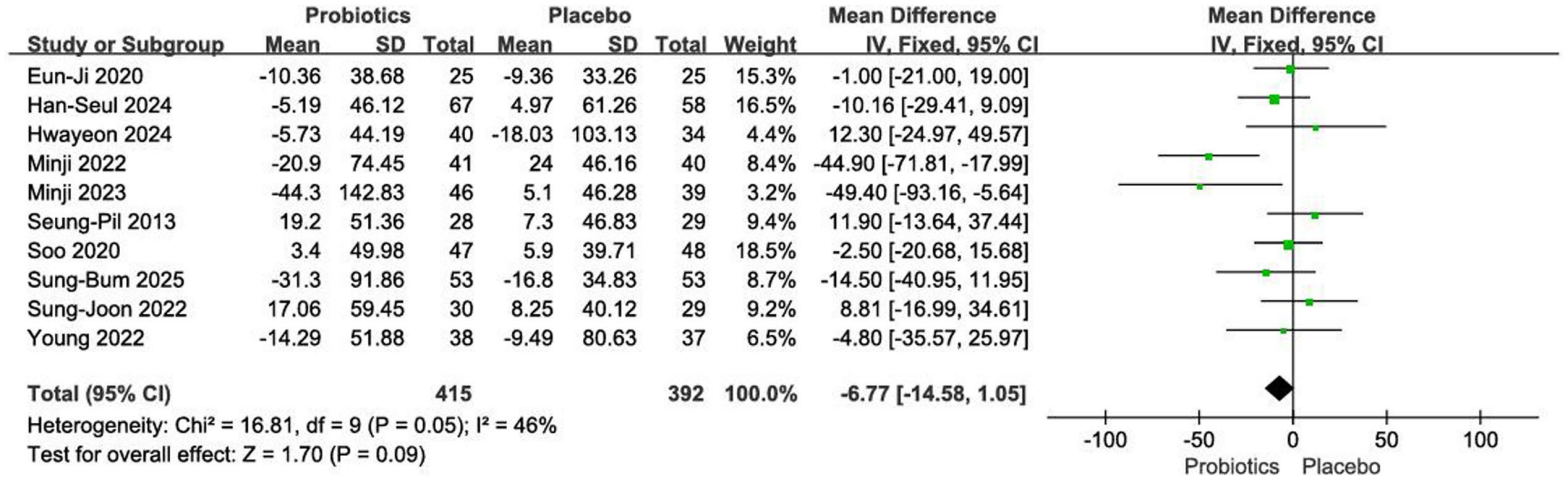
Forest plot of meta-analysis on triglyceride.

Ten RCTs comprising 807 participants were included in this analysis (12, 18, 19, 21–28). Given the moderate between-study heterogeneity (*I*^2^ = 46%, *p* = 0.05), a fixed-effects model was applied. The pooled results showed no significant difference in lipid reduction between the probiotic and placebo groups [MD = −6.77 mg/dL, 95% CI (−14.58, 1.05), *p* = 0.09] (Figure 9).

### 3.5 Subgroup analysis results

To explore the intervention effects on body weight across different subgroups, subgroup analyses were conducted on potential moderator variables to determine the pooled effect sizes for each subgroup. The subgroup analysis results demonstrate that distinct elements of the intervention regimens had differential impacts on body weight metrics in obese patients. The findings of the subgroup analysis are displayed in Table 2. The single-strain subgroup showed a greater reduction in body weight (SMD = −0.90) than the multi-strain subgroup (SMD = −0.27). The between-subgroup difference approached statistical significance (*χ*^2^ = 2.48, *p* = 0.12), compared to the *p*-values for differences among other subgroups, the p-value for this subgroup difference is relatively closer to the threshold of statistical significance; A significant weight-reducing effect was observed in the high-dose subgroup (SMD = −0.51), whereas the effect in the low-dose subgroup was non-significant (SMD = −0.60). The difference between dose subgroups was not statistically significant (*χ*^2^ = 0.01, *p* = 0.92); the probiotic intervention plus health guidance resulted in a larger weight reduction (SMD = −0.89) compared with the probiotic-only intervention (SMD = −0.28). The between-subgroup difference approached statistical significance (*χ*^2^ = 2.32, *p* = 0.13); A significant effect was found in the simple obesity subgroup (SMD = −0.51) but not in the overweight/obesity subgroup (SMD = −0.94). The difference between population subgroups was not statistically significant (*χ*^2^ = 0.13, *p* = 0.72).

**Table 2:** Subgroup analysis of the effect of probiotics on weight intervention in obese patients.

Subgroup	Number of included studies (articles)	Heterogeneity test result		Effect model	Meta-analysis result		Test for subgroup differences	
		*I*^2^ value (%)	*p*-value		OR(95%CI)	*p*-value	*x* ^2^	*p*-value
Inclusion population							0.13	0.72
Simple obesity	8 (12, 18, 23, 25–29)	0	0.88	Fixed	−0.51 [−0.90, −0.21]	0.01		
Overweight/obesity	3 (20, 21, 24)	0	0.88	Fixed	−0.94 [−3.32, 1.44]	0.44		
Intervention dose							0.01	0.92
High-dose (≥1 × 10^10^ per day)	8 (18, 21, 23–25, 27, 29)	0	0.88	Fixed	−0.51 [−0.91, −0.21]	0.01		
Low-dose (<1 × 10^10^ per day)	3 (12, 20, 26)	0	0.82	Fixed	−0.60 [−2.22, 1.02]	0.47		
Number of strains							2.48	0.12
Single-strain	7 (12, 20, 21, 23, 25, 26, 28)	0	1.00	Fixed	−0.90 [−1.52, −0.29]	0.004		
Multi-strain	4 (18, 24, 27, 29)	0	0.94	Fixed	−0.27 [−0.76, 0.22]	0.28		
Intervention method							2.32	0.13
Probiotic-only intervention	5 (20, 21, 24, 27, 29)	0	0.97	Fixed	−0.28 [−0.77, 0.22]	0.27		
Probiotic intervention plus health guidance	6 (12, 16, 18, 23, 25, 26, 28)	0	0.99	Fixed	−0.89 [−1.51, 0.28]	0.005		

### 3.6 Publication bias and sensitivity analysis results

**Figure 10:**
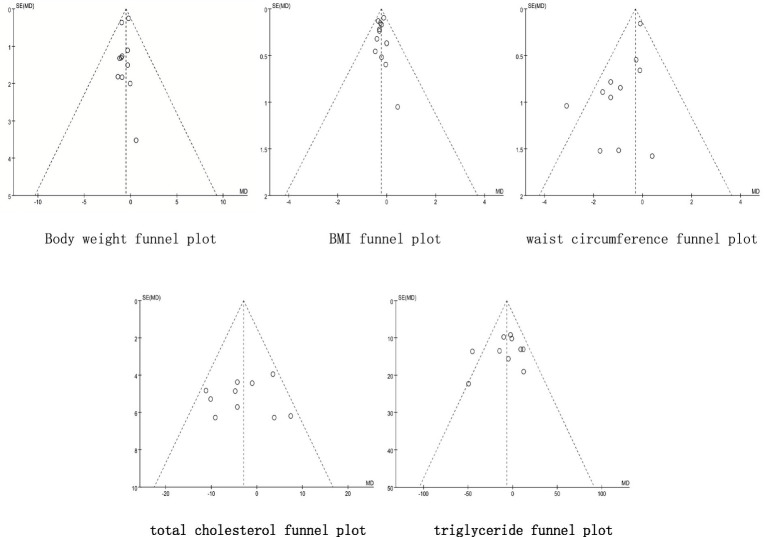
Publication bias funnel plot.

**Figure 11:**
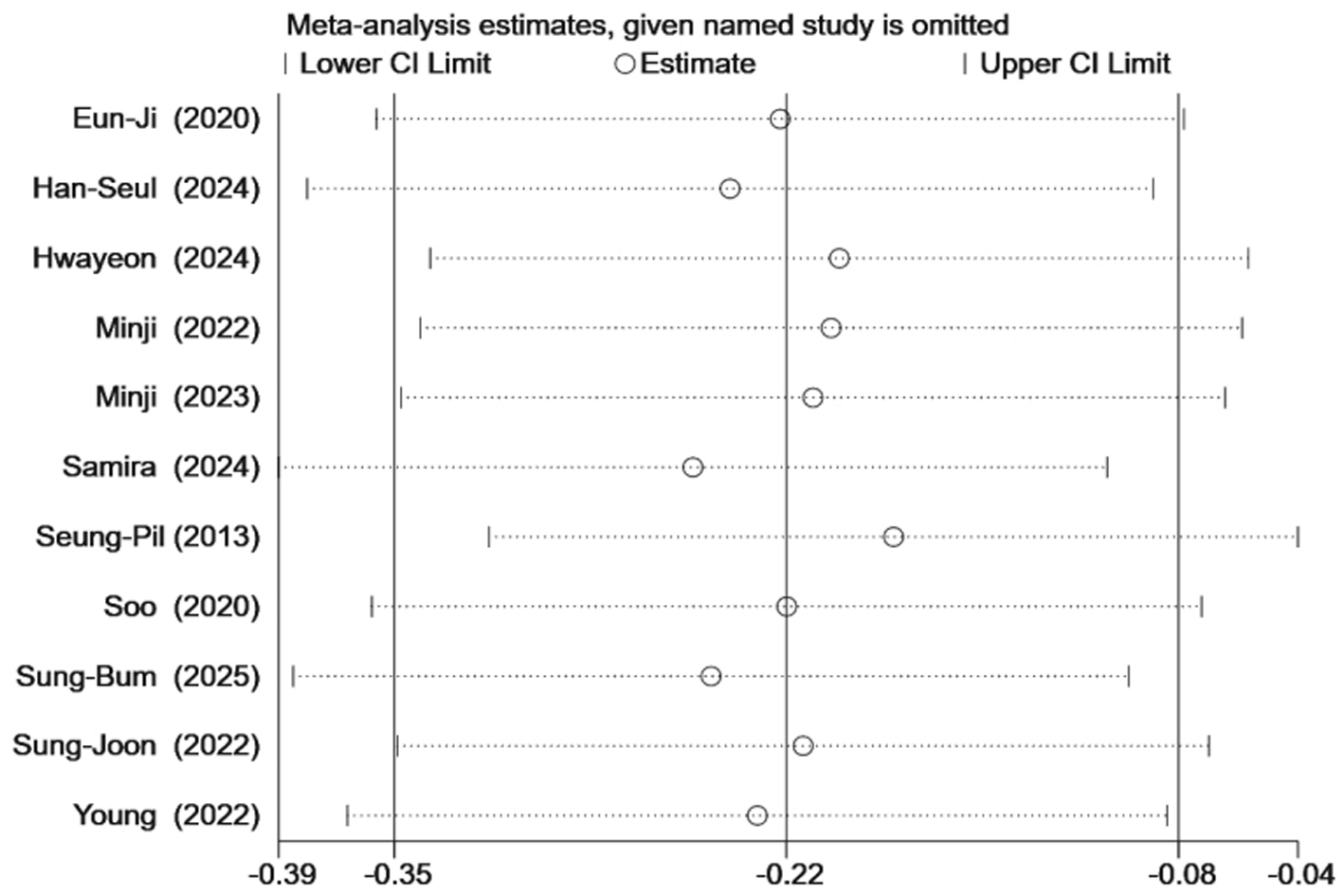
Waist circumference sensitivity analysis chart.

Publication bias was assessed using funnel plots and Egger’s test for outcomes with 10 or more studies (body weight, BMI, waist circumference, total cholesterol, and triglycerides): The funnel plots for total cholesterol and triglycerides were symmetrical, whereas asymmetry was observed for body weight, BMI, and waist circumference (Figure 10). Egger’s test indicated no significant publication bias for weight (*p* = 0.782) and BMI (*p* = 0.354), but significant bias was detected for waist circumference (*p* = 0.041). The trim-and-fill procedure did not materially change the results for waist circumference, supporting the robustness of the initial findings. Sensitivity analysis was conducted by sequentially excluding individual studies (Figure 11). The overall pooled effect sizes remained stable, confirming that the main conclusions were not substantially influenced by any single study. Taken together, although the existence of publication bias in this study, the findings of this meta-analysis are considered robust.

## 4 Discussion

### 4.1 Key findings and public health implications

This meta-analysis synthesized evidence from 12 randomized controlled trials involving 931 individuals with obesity to evaluate the effects of probiotic supplementation on weight management. The pooled results indicated that probiotic intervention significantly improved body weight, body mass index, waist circumference, and body fat percentage. In contrast, no statistically significant effects were observed on lipid profiles (total cholesterol and triglycerides). The findings indicate that probiotics are not universally helpful for all obesity-related parameters. The efficacy of probiotic intervention appears to be influenced by factors such as baseline participant characteristics, number of strains, and dosage, underscoring the need for tailored approaches. All interventions had a favorable safety profile. The effects of probiotics on obesity-related indicators are outcome-specific. To assess their value, consider baseline subject characteristics, strain composition, and dosage. Current evidence shows probiotic supplementation is a useful nutritional adjunct. For individuals needing significant improvement in lipid metabolism, include other interventions in the management plan. Future directions should focus on developing combined formulations with synergistic mechanisms and clear advantages to enhance probiotics’ application in comprehensive obesity management.

### 4.2 Strain specificity

#### 4.2.1 Functional divergence between core genera

While probiotic interventions demonstrate positive significance at the population level, the heterogeneity in outcomes across different primary studies points to a deeper scientific reality: distinct genera (and even different strains within the same genus) may exert differential physiological effects through unique mechanisms of action. This study’s probiotic interventions primarily focus on the genera *Lactobacillus* and *Bifidobacterium*. The original study designs are limited and prevent a direct comparison of efficacy differences between these two genera. However, integrating existing evidence with mechanistic research helps outline their potentially distinct pathways of action. This offers a key perspective for understanding the heterogeneous effects of probiotics. The role of Lactobacillus may be more focused on improving intestinal barrier function and modulating local/systemic inflammation. It can enhance the expression of tight junction proteins, reduce metabolic endotoxemia, and thereby alleviate chronic low-grade inflammation associated with obesity (30), which is particularly crucial for reducing visceral fat accumulation; the core advantage of Bifidobacterium likely lies in its efficient fermentation of dietary fiber to produce Short-Chain Fatty Acids (SCFAs), particularly acetate and butyrate. SCFAs are not only an energy source for the host but also serve as important signaling molecules. They can regulate the secretion of appetite-related hormones, enhance fat oxidation, and inhibit fat synthesis by activating G protein-coupled receptors (31). Therefore, Bifidobacterium intervention may play a more direct role in modulating energy intake and improving insulin sensitivity.

#### 4.2.2 Interventional efficacy advantage of single strains

Subgroup analysis indicated that the weight-reducing effect of single-strain intervention was superior to that of multi-strain combinations. Compared with the *p*-values of differences in other subgroups, the *p*-value for this group difference (*p* = 0.12) was numerically lower than those of other subgroups (e.g., dose: *p* = 0.92; method: *p* = 0.13), suggesting that “tnumber of strains” may be a relatively more influential moderating factor worthy of further investigation in larger-scale studies. This finding also suggests that in probiotic interventions, the intrinsic efficacy of a specific strain may be more critical than merely increasing the number of strains, further highlighting the importance of identifying “strain specificity.” However, some studies have reported the opposite result, suggesting that multi-strain combinations may be more effective in improving anthropometric measures (32, 33). Such discrepancies may stem from synergistic interactions, complementary mechanisms among strains, or systemic modulation of the gut microbiota. Therefore, future probiotic research must move beyond superficial discussions on “number of strains” and delve into the alignment of strain identity, functional mechanisms, and host characteristics to achieve truly precise interventions.

#### 4.2.3 Parameters defining precision research

Based on the results of subgroup analysis, future studies aimed at promoting the precise application of probiotics must strictly define and control the following key parameters. First, the dose subgroup results identified an “effective threshold” of ≥1 × 10^10^ CFU/day, which should serve as a reference for designing future studies. Second, the “probiotic intervention plus health guidance” group demonstrated significantly superior weight loss effects compared to the probiotic-only intervention group, revealing the potential of a “microecology-behavior” synergistic strategy. This suggests that effective strain interventions can create a more favorable physiological foundation for lifestyle modifications. Additionally, the subgroup analysis highlighted the importance of population specificity. This study found that probiotic interventions showed a more pronounced weight loss trend in individuals with simple obesity. This indicates that the effects of probiotics may be influenced by obesity type, baseline metabolic status, and gut microbiota characteristics. Therefore, future precision research needs to further clarify the differential effects of probiotics across various obesity subtypes and establish population-stratified intervention strategies by incorporating biomarkers such as microbiome characteristics. This will provide empirical evidence for developing scalable and sustainable obesity management programs.

### 4.3 The effect of probiotics on lipid metabolism indicators is not significant

In this meta-analysis, no statistically significant differences were seen between the probiotic group and the placebo group regarding the two primary lipid outcomes: total cholesterol and triglycerides. This finding contrasts with certain prior research, such as the RCT conducted by Minji et al. (26), which reported significant reductions in total cholesterol and triglyceride levels following Lactobacillus supplementation in individuals with obesity. Potential explanations for this inconsistency encompass: (1) Notable discrepancies in probiotic strains employed in various trials. This investigation encompassed strains from the Lactobacillus, Bifidobacterium, and Weissella genera, with distinct strains potentially demonstrating significant variations in their lipid-regulating capacities. Brusaferro et al. (34) observed that the effects of probiotics on body weight and metabolic indicators are “strain-specific.” Certain strains may focus on body weight rather than blood lipids, and this variability may have diminished the aggregated impact size; (2) The majority of the research examined utilized 12-week intervention durations. Regulation of lipid metabolism may necessitate increased dosages or prolonged intervention durations. These co-interventions may have obscured or disrupted the effects of probiotics on lipids. (3) The baseline blood lipid levels of participants differed among research studies, with some individuals potentially classified as being in a “high-normal” range. Probiotics may exhibit enhanced efficacy in populations with significantly aberrant lipid levels, and this baseline heterogeneity may have influenced the lack of significance in the aggregated study. Therefore, existing evidence is inadequate to endorse probiotic supplementation as a public health policy aimed at enhancing population lipid levels. Future public-health-oriented research should prioritize large-scale community trials that examine specific probiotic strains, longer intervention periods, and cost-effectiveness to inform evidence-based policy.

### 4.4 Public health strategies and policy recommendations

Realizing the public health value of probiotics hinges on enhancing population accessibility and implementation feasibility. The availability of probiotics in various formats, such as capsules, as well as widely consumed foods like yogurt and fermented beverages, offers inherent advantages for large-scale interventions. This aligns with the principles of ‘low-cost, high-adherence’ public health strategies (35). Promotion and integration efforts could focus on channels such as community health centers and clear product labeling guidelines to facilitate the incorporation of probiotic options into individuals’ daily dietary choices. This approach is well-suited for integration into community-based population health management and chronic disease prevention programs.

However, to achieve precise promotion of probiotics at the public health level, it is essential to address the core challenge revealed by this study: the high uncertainty of strain-specific effects. Therefore, future public health research must undergo a paradigm shift from “category effectiveness” to “strain effectiveness,” prioritizing high-quality, large-scale, multicenter randomized controlled trials targeting specific high-quality strains. These studies should aim to clarify their exact efficacy and cost-effectiveness across different populations and intervention scenarios. On this basis, policy development should prioritize three key areas: First, establish scientific criteria for probiotic strain approval, including specifications for applicable strains, intervention duration, and dosage. Second, reduce production costs through measures such as fiscal subsidies to promote the widespread adoption of probiotic-fortified foods. Third, explore the gradual inclusion of interventions involving specific strains with clear evidence-based support into budgets for chronic disease prevention and health management. To support these initiatives, further high-quality research is needed on cost-effectiveness and safety, accumulating evidence through community pilot programs to provide a basis for future large-scale implementation (35).

Promoting probiotic interventions into the primary prevention phase of obesity carries important public health value. Studies have shown that probiotics can notably enhance weight-related indicators in obese individuals without dietary restrictions, while also generating long-term health benefits through early-life gut microbiota modulation (36). Thus, probiotics represent a key approach to primary obesity prevention, suitable for application in healthy populations, high-risk overweight groups (e.g., individuals with a family history of obesity or sedentary lifestyles), and critical early stages, including infancy, childhood, and adolescence.

### 4.5 Limitations

Several limitations of this meta-analysis warrant consideration when interpreting the results. First, regarding methodological limitations, despite employing a comprehensive search strategy, some studies may have been missed, and publication bias cannot be ruled out. Second, the nature of the included evidence is limited, as most RCTs were small, single-center trials with limited statistical power. Non-significant interaction tests in subgroup analyses may reflect Type II error. Third, substantial clinical and methodological heterogeneity was observed, with notable variations in probiotic strains, formulations, and dosing regimens. Although subgroup analyses were conducted, inferences regarding “probiotics” as a class should be made cautiously. Fourth, although the probiotic effects observed in this study primarily originate from the genera *Lactobacillus* and *Bifidobacterium*, most of the original studies did not include comparative arms of different genera within the same trial. As a result, we are unable to directly quantify and compare the efficacy differences between *Lactobacillus* and *Bifidobacterium* in obesity management through meta-analysis. Therefore, the discussion regarding these two genera in this paper is largely based on mechanistic reasoning and data inference, the certainty of which requires validation through future direct comparative studies. Fifth, the scope and duration of outcome measures were limited. All trials lasted 12 weeks, precluding comparisons of effects across different intervention periods. Fifth, the generalizability of findings is constrained. Studies were highly concentrated in East Asia (11/12) and participants shared a diet rich in fermented foods, raising questions about applicability to populations with markedly different baseline gut microbiota. The positive effects of specific dietary interventions may linked to this unique ecological context. In light of these limitations, the overall certainty of evidence is moderate, indicating that future research could alter the current conclusions and that generalizability should be interpreted with caution.

## 5 Conclusion

While numerous probiotic studies exist, most are single-center trials with limited sample sizes. The present meta-analysis clarifies the significant role of probiotics in weight management for individuals with obesity, showing that probiotic intervention significantly reduced body weight, BMI, waist circumference, and body fat percentage. No adverse events were reported across included studies, indicating a favorable safety profile. However, no significant improvements were observed in lipid metabolism markers, such as total cholesterol and triglycerides, suggesting that the therapeutic effects of probiotics may be outcome-specific and should be applied selectively based on clinical goals. Subgroup analysis further revealed that among individuals with obesity alone, probiotic intervention protocols featuring a single strain, high dosage (≥1 × 10^10^ CFU/day), and combined health guidance demonstrated superior weight loss outcomes, providing an evidence-based framework for designing probiotic regimens in clinical and public health practice. It is particularly noteworthy that the synthesized evidence in this study primarily stems from interventions involving the genera *Lactobacillus* and *Bifidobacterium*. The analysis suggests that these two genera may influence obesity-related indicators through distinct core mechanisms, providing an important theoretical basis for the preliminary selection of probiotics tailored to specific patient phenotypes in the future.

Future endeavors to actualize the potential of probiotics in obesity prevention and management should concentrate on: in scientific research, research should be expanded to investigate strain specificity, population adaptability, and mechanisms of action, including conducting high-quality comparative trials of strains from the genera Lactobacillus and Bifidobacterium in obesity management. This will help clarify the similarities and differences in their effects and establish a more solid theoretical foundation for precise interventions; In public health practice, probiotics should shift from being “clinical adjuncts” toward “primary prevention” strategies, integrating them gradually into community health management and targeted population care. Public education and contextualized promotion can enhance acceptance and adherence. At the policy and application level, urgent efforts are needed to develop an evidence-based standard system for probiotic interventions. In parallel, collaboration with the food industry—guided by public health priorities—can improve the accessibility of probiotic products for those who may benefit. Ultimately, an integrated “research–practice–policy” approach could position probiotics as a sustainable component of comprehensive strategies to prevent and manage obesity, thereby contributing to global efforts to reduce the burden of obesity-related chronic diseases.

## Data Availability

The original contributions presented in the study are included in the article/supplementary material, further inquiries can be directed to the corresponding authors.
