# Supplementary material for: Efficacy of probiotic supplementation for body weight management in overweight and obese adults: a meta-analysis of randomized controlled trials predominantly from East Asia
Source: Front Public Health. 2026 Mar 20;14:1767108. doi: 10.3389/fpubh.2026.1767108 (PMC13046508; doi:10.3389/fpubh.2026.1767108)
Supplement: Supplementary file 1 [file Table_1.docx]

**Supplementary Table S1**

**Search formulas**

| 1. Pubmed | | |
| --- | --- | --- |
| #1 | Search: "Probiotics"[Mesh] Sort by: Most Recent |  |
| #2 | Search: Probiotic[Title/Abstract] |  |
| #3 | #1 OR #2 |  |
| #4 | Search: "Obesity"[Mesh] Sort by: Most Recent |  |
| #5 | Search: (overweight[Title/Abstract]) OR (obese[Title/Abstract]) Sort by: Most Recent |  |
| #6 | #4 OR #5 |  |
| #7 | Search: "Body Weight"[Mesh] Sort by: Most Recent |  |
| #8 | Search: ((Body Weights[Title/Abstract]) OR (Weight, Body[Title/Abstract])) OR (Weights, Body[Title/Abstract]) Sort by: Most Recent |  |
| #9 | #7 OR #8 |  |
| #10 | #3 AND #6 AND #9 | 1072 |

| 2. Embase | |  |
| --- | --- | --- |
| #1 | 'probiotic agent'/exp OR 'probiotic agent' OR probiotic:ti,ab,kw OR probiotics:ti,ab,kw OR 'probiotic agent':ti,ab,kw |  |
| #2 | 'obesity'/exp OR 'obesity' OR 'adipose tissue hyperplasia':ti,ab,kw OR adipositas:ti,ab,kw OR adiposity:ti,ab,kw OR 'alimentary obesity':ti,ab,kw OR 'body weight, excess':ti,ab,kw OR corpulency:ti,ab,kw OR 'fat overload syndrome':ti,ab,kw OR 'nutritional obesity':ti,ab,kw OR obesitas:ti,ab,kw OR overweight:ti,ab,kw |  |
| #3 | 'weight'/exp |  |
| #4 | #1 AND #2 AND #3 | 1044 |

| 3. Web of Science | |  |
| --- | --- | --- |
| #1 | Probiotic (Topic) or Probiotics (Topic) |  |
| #2 | obesity (Topic) or obese (Topic) or overweight (Topic) |  |
| #3 | Body Weight (Topic) or Body Weights (Topic) or Weight, Body (Topic) or Weights, Body (Topic) |  |
| #4 | #1 AND #2 AND #3 | 1008 |

| 4. Cochrane Library | | |
| --- | --- | --- |
| #1 | MeSH descriptor: [Probiotics] explode all trees |  |
| #2 | (Probiotic):ti,ab,kw |  |
| #3 | #1OR#2 |  |
| #4 | MeSH descriptor: [Obesity] explode all trees |  |
| #5 | MeSH descriptor: [Body Weight Changes] explode all trees |  |
| #6 | #3AND#4AND#5 | 45 |

| 5.知网 | | |
| --- | --- | --- |
|  | （主题：益生菌）OR（主题：probiotics+微生态制剂+活菌制剂+益生素+有益菌+有益微生物）AND（主题：肥胖）OR（主题：超重）AND（主题：体重）OR（主题：BMI+体脂+体脂率+腰围+减重） | 69 |

| 6.万方 | | |
| --- | --- | --- |
|  | 主题:(益生菌 or probiotics or 微生态制剂 or 活菌制剂 or 益生素 or 有益菌 ) and 主题:(肥胖 or 超重) and 主题:(体重 or BMI or 体脂 or 体脂率 or 腰围 or 减重) | 988 |

| 7.维普 | | |
| --- | --- | --- |
|  | 题名或关键词=益生菌+probiotics+微生态制剂+活菌制剂+益生菌剂+益生素+有益菌+有益微生物AND题名或关键词=肥胖+超重AND题名或关键词=体重+BMI+体脂+体脂率+腰围+减重 | 7 |

### Supplementary Table S2

### Cochrane Risk of Bias Assessment Tool (RoB 1.0) Predefined Judgment Criteria

### 1.Random sequence generation (selection bias)

| **Risk Level** | **Predefined Judgment Criteria** |
| --- | --- |
| Low risk | Clearly describe randomization methods (e.g., random number table method, computer-generated randomization, stratified randomization, block randomization), ensuring scientific rigor and reproducibility to guarantee baseline comparability between groups (e.g., mention balanced baseline indicators such as BMI, age, and gender). |
| High risk | Non-random assignment methods (e.g., by birth date, last digit of hospital admission number, order of presentation, or investigator's subjective selection); or described as “random” without specifying the method (e.g., merely stating “random assignment” without details). |
| Unclear risk | The random sequence generation method is not specified; or the description is vague (e.g., “using a random method” without operational details), making it impossible to assess the scientific validity of the approach. |

### 2.Allocation concealment (selection bias)

| **Risk Level** | **Predefined Judgment Criteria** |
| --- | --- |
| Low risk | Clearly describe methods for concealing allocation (such as central randomization, sealed opaque envelopes, or telephone/web-based random assignment systems) to prevent researchers or participants from learning their group assignment in advance. |
| High risk | Did not employ allocation concealment (e.g., using open random allocation tables, non-sealed/transparent envelopes); or used a predictable allocation method (e.g., alternating allocation, grouping by bed number). |
| Unclear risk | No mention of the concealment method used; or the description is vague (e.g., “using the envelope method” without specifying whether it is sealed/opaque), making it impossible to determine if effective concealment was achieved. |

**3.Blinding of participants and personnel (performance bias)**

| **Risk Level** | **Predefined Judgment Criteria** |
| --- | --- |
| Low risk | Both subjects and investigators were blinded (double-blind), with detailed descriptions of the blinding implementation (e.g., probiotics and placebo were identical in formulation, appearance, odor, and administration method; researchers were not involved in group/intervention assignment); if researcher blinding was not feasible due to intervention characteristics (e.g., probiotics administered as specific food formulations), but participant blinding was clearly maintained, and it was stated that unblinded researchers did not influence outcome assessments (e.g., weight and BMI as objective measures). |
| High risk | Unblinded (e.g., when subjects/investigators explicitly know their group assignment); or incomplete blinding (where only one party is blinded, and the unblinded party may influence intervention implementation or subject compliance, such as when investigators proactively adjust subjects' dietary/exercise regimens); or unmasking (e.g., when subjects experience pronounced adverse reactions that allow inference of their group assignment). |
| Unclear risk | The study did not mention blinding; or the description was vague (e.g., “blinded” without specifying the subjects or methods); or it was unclear whether the blinding was broken. |

### 4.Blinding of outcome assessment (detection bias)

| **Risk Level** | **Predefined Judgment Criteria** |
| --- | --- |
| Low risk | Blinding of outcome assessors (e.g., assessors not involved in randomization/intervention and unaware of subject assignment) or use of objective outcome measures (e.g., weight, BMI, waist circumference measured via electronic scales/tape measures; body fat percentage assessed by instrument) with standardized measurement protocols (e.g., consistent timing, calibrated tools) ensures that non-blinded assessors do not compromise outcome objectivity. |
| High risk | The outcome assessors were aware of the subjects' group assignments, and the outcome measures contained subjective judgment components (e.g., “degree of body fat improvement” and “dietary adherence” relied on assessors' subjective scoring); or the measurement methods for objective indicators were not standardized (e.g., different measurement tools were used across groups, or tools were not calibrated). |
| Unclear risk | The study did not specify whether outcome assessors were blinded; or descriptions were ambiguous (e.g., “objectively measured outcomes” without clarifying assessor knowledge of group assignment); or subjective measures failed to indicate assessor blinding status. |

**5.Incomplete outcome data (attrition bias)**

| **Risk Level** | **Predefined Judgment Criteria** |
| --- | --- |
| Low risk | Report the total number of participants lost to follow-up in each group and the reasons for loss (e.g., loss to follow-up, withdrawal due to adverse events, voluntary withdrawal). The loss to follow-up rate should be ≤20% (for short-term interventions ≤12 weeks) or ≤15% (for long-term interventions >12 weeks), with balanced rates between groups (difference ≤5%). Apply appropriate methods for handling missing data. |
| High risk | Loss to follow-up rate > 20% (short-term) or > 15% (long-term); or intergroup loss to follow-up rate difference > 5% without explanation; or missing data not handled (e.g., using only per-protocol set analysis for PP while excluding large numbers of lost-to-follow-up participants); or reasons for loss to follow-up related to outcomes (e.g., intervention group dropouts due to lack of weight improvement). |
| Unclear risk | Number of unreported missing cases / reasons; or vague descriptions of missing case rates (e.g., “a few missing cases”); or failure to explain methods for handling missing data. |

### 6.Selective reporting (reporting bias)

| **Risk Level** | **Predefined Judgment Criteria** |
| --- | --- |
| Low risk | All primary outcome measures specified in advance in the study protocol (e.g., registration information, baseline documentation)—including weight, BMI, waist circumference, and body fat percentage—have been reported. No significant omissions were identified in the secondary outcome measures. Statistical methods were consistent with pre-specified plans (e.g., the pre-specified analysis using mean difference [MD] was applied without modification). |
| High risk | Failure to report pre-specified primary outcome measures (e.g., reporting only BMI without weight/waist circumference); selective reporting of favorable results (e.g., reporting only significant weight loss in the intervention group while concealing no difference in body fat percentage); or altering outcome measure definitions/statistical methods (e.g., prescribing “weight change value” but reporting “weight attainment rate”). |
| Unclear risk | No study protocol available for comparison (e.g., unregistered, no mention of pre-specified outcomes); or outcome reporting is incomplete (e.g., only outcome names listed without specific data), making it impossible to determine whether selective reporting occurred. |

### **7.**Other bias

| **Risk Level** | **Predefined Judgment Criteria** |
| --- | --- |
| Low risk | No significant other confounding factors: such as balanced baseline data (comparable BMI, age, gender, etc., under Asian obesity standards); clearly defined probiotic intervention details (consistent strains, dosage, intervention duration, and formulation); no conflicts of interest declared (or declaration of no relevant conflicts of interest); study not terminated prematurely (or premature termination justified with complete data). |
| High risk | Clear sources of bias exist: - Severe baseline imbalances (e.g., significantly higher mean BMI in the intervention group compared to the control group, without adjustment); - Unclear details of probiotic intervention (e.g., failure to specify strain types, dosage, or changes to the intervention protocol mid-study); - Conflict of interest (e.g., researchers receiving funding from probiotic manufacturers without disclosure); - Premature study termination without explanation (e.g., discontinuation due to poor efficacy without reporting complete data). |
| Unclear risk | Unable to determine whether other biases exist: - Partial missing baseline data; - Vague description of probiotic intervention details; - No mention of conflicts of interest; - Potential issues in study design but insufficient information for verification (e.g., small sample size without justification). |
